# Severe Clozapine-Induced Agranulocytosis, Pharmacodynamic Synergism, and Lithium Promyelocytic Effects in Granulocyte Colony-Stimulating Factor (G-CSF) Resistance or Delayed Response: A Case Report

**DOI:** 10.7759/cureus.52092

**Published:** 2024-01-11

**Authors:** Philipa Owusu-Antwi, Edmund Appiah-Kubi, Meheret Kinfe, Sarah Gbinigie, Archna Sarwal

**Affiliations:** 1 Psychiatry, Richmond University Medical Center, Staten Island, USA; 2 Internal Medicine, Allegheny Health Network, Pittsburgh, USA; 3 Psychiatry, American University of Antigua, Staten Island, USA; 4 Psychiatry, St. George's University School of Medicine, St. George's, GRD

**Keywords:** antipsychotics, promyelocytosis, lithium, drug induced agranulocytosis, clozapine

## Abstract

Clozapine-induced agranulocytosis is documented in multiple studies, but there is limited literature on treatment-resistant agranulocytosis, the synergistic effect of multiple agranulocytosis-inducing drugs, and lithium's promyelocytic effects. This case highlights these gaps by discussing a patient with an absolute neutrophil count of zero while being treated with Depakote, Haldol, and clozapine. This case proposes more research on lithium's promyelocytic effects, especially in patients who are unresponsive or have a delayed response to discontinuing offending agents and granulocyte colony-stimulating factor (G-CSF).

## Introduction

Clozapine, a highly effective antipsychotic used in the management of treatment-resistant schizophrenia, is limited in its use due to the risk of agranulocytosis; a rare condition characterized by an absolute neutrophil count (ANC) of less than 500 cells/mm³, and occurs in up to 0.4% of patients receiving clozapine [1-3]. While clozapine-induced agranulocytosis (CIA) itself poses a significant clinical challenge, instances of treatment-resistant cases further compound the complexity of managing this condition, characterized by a lack of response to standard therapeutic interventions. These include discontinuation of the offending drug, administration of granulocyte colony-stimulating factor (G-CSF), and broad-spectrum antibiotics [2]. The efficacy of G-CSF in the management of drug-induced neutropenia is well documented [4]. While lithium has been observed to elevate neutrophil counts, the use of lithium as an adjunctive therapy has not been thoroughly explored [5]. This report presents a patient with a diagnosis of CIA, who despite conventional interventions continued to experience a decline in neutrophil count. The unexpected rebound leukocytosis following G-CSF cessation, in addition to the introduction of lithium as an adjunct, adds a novel dimension to our understanding of the management of CIA.

## Case presentation

A 70-year-old Caucasian female diagnosed with chronic schizophrenia, residing at a long-term psychiatric care facility presented to the hospital with complaints of minimal generalized weakness and was admitted for evaluation of severe leukopenia. The patient had no other complaints. The patient's review of systems and physical examination was unremarkable except for prior localized pain. The patient was recently started on clozapine and titrated to 300 mg oral (PO) daily in the month before admission after a 12.5 mg PO trial. The patient received olanzapine 5mg intramuscularly, twice a day (IM, BID) if the patient declined oral dose per court order. The patient was also treated with haloperidol 5 mg PO at bedtime for which she received haloperidol 2.5 mg IM she refused oral dose per court order. The patient also received haloperidol decanoate 200 mg IM every four weeks for a year, and divalproex sodium ER 500 mg PO BID for a year. The patient was court-ordered to receive haloperidol 5 mg IM BID if the patient declined the oral dose of divalproex sodium. The patient was compliant with oral medications. Her last haloperidol decanoate dose was 18 days prior to admission, with the next dose held during admission due to leukopenia. Clozapine was held two days prior to admission and discontinued on the day of admission.

On admission, she was afebrile (99.0°F), her blood pressure was 126/68 mmHg, her pulse was 98 bpm, and her respiratory rate was 18 cycles per minute with an oxygen saturation of 98% on room air. A complete blood count (CBC) showed a white blood cell count (WBC) of 0.6 103/µL and an ANC of 0.02 103/µL. The chest x-ray, urine, and blood cultures were negative. She was diagnosed with drug-induced neutropenia secondary to clozapine use and was started on G-CSF 480 mcg once daily. On day 2 of hospitalization, she became febrile with a temperature of 102.9°F and developed a cough with minimal sputum, but did not meet the criteria for sepsis. She was treated with intravenous vancomycin, aztreonam, and cefepime for neutropenic fever with no apparent source of infection. Serial blood, urine, sputum cultures, and viral respiratory panels were negative. Serial chest X-rays were also negative. By day 3, the patient's fever had resolved.

**Figure 1:**
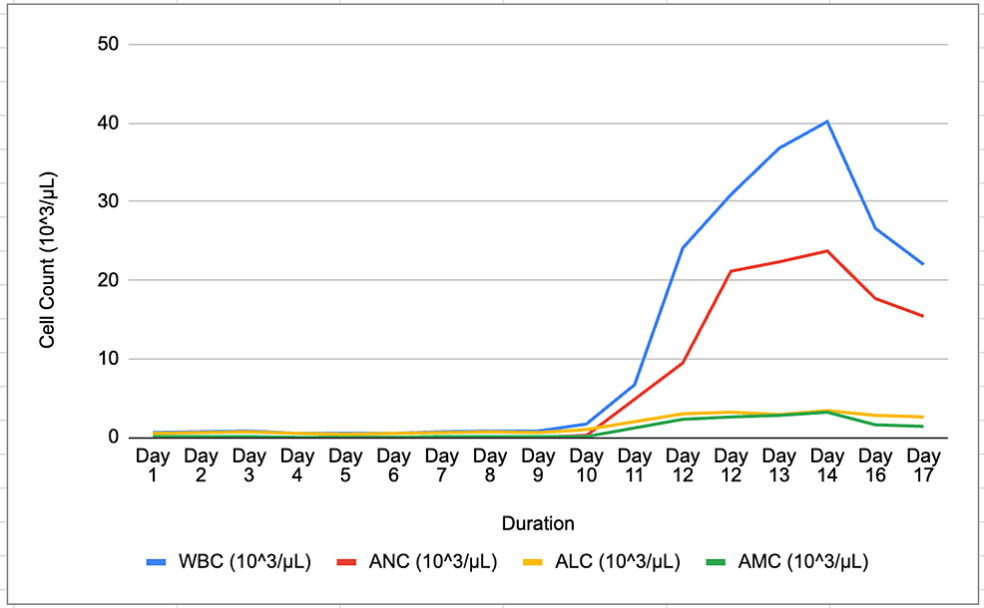
Demonstrates the progression and resolution of neutropenia Figure was created by authors. WBC: white blood cell count; ANC: absolute neutrophil count; ALC: absolute lymphocyte count; AMC: absolute monocyte count

Upon psychiatric evaluation, it was recommended to hold haloperidol and decrease the dose of divalproex sodium due to the synergistic effects of clozapine, haloperidol, and divalproex sodium in precipitating leukopenia. It was recommended on day 4 of admission to hold divalproex sodium if it was not prescribed as an anti-seizure medication and to consider using lithium's promyelocytic effects as an adjunct, and for mood stabilization. Lithium carbonate 300 mg twice daily was started on day 6 to augment GCS-F due to the slow response to G-CSF monotherapy in persisting agranulocytosis with a lower ANC than the ANC on admission. As shown in Figure 1, CBC was monitored daily, exhibiting neutropenia until day 11, with a WBC of 24.1 103/µL and ANC of 9.47 103/µL. Leukocytosis was believed to be due to G-CSF and adjunct lithium therapy. G-CSF was discontinued on day 10. Divalproex sodium 500 mg twice daily was discontinued on day 11, and lithium was continued as the preferred mood stabilizer as the patient's kidney function and thyroid function tests were within normal limits.

**Figure 2:**
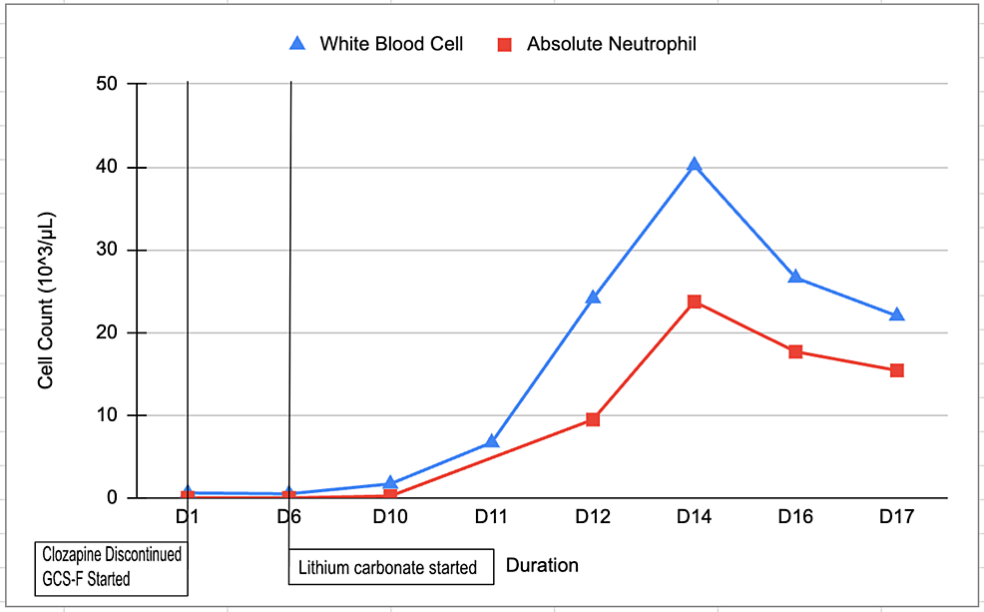
Demonstrates the effects of clozapine cessation, G-CSF treatment, and adjunct lithium use on WBC and ANC Figure was created by authors. G-CSF: granulocyte colony-stimulating factor; WBC: white blood cell count; ANC: absolute neutrophil count

The patient's laboratory test before discharge CBC showed WBC 22.0 103/µL and ANC 15.41 103/µL as seen in Figure 2. The patient was discharged with close instructions to follow up regularly with the primary care doctor and psychiatrist for continuous monitoring and management.

## Discussion

Clozapine, the FDA-approved and most effective medication for treatment-resistant schizophrenia (TRS), is underutilized due to monitoring and tolerability concerns, which can potentially increase mortality due to CIA [6]. Providers are required to enroll and be certified in a safety program by the FDA known as Clozapine REMS (Risk Evaluation and Mitigation Strategy) to monitor patients taking clozapine. The standard practice according to FDA Clozapine REMS guidelines is to monitor patients with weekly complete blood count (ABC) for the first 6 months, then bi-weekly for the next 6 months. If a patient is compliant with blood work and ANC has been normal, CBC is recommended monthly after the first year of taking clozapine [2].

Early treatment with clozapine improves response compared to delayed use after failed medication trials and severe TRS [7]. Initiating clozapine earlier decreases hospitalizations, lowering healthcare costs [8]. Islam et al. found that people with the HLA-DRB1*04:02 allele had nearly sixfold (95% CI 2.20, 15.80) greater odds of CIA with a high NPV 99.3%, suggesting that it may be useful in stratifying individuals at risk of CIA [9]. It is important for clinicians to be cautious when using multiple agranulocytosis-inducing drugs. The patient in our case report was treated with Haldol and Depakote for a year without any major side effects until the addition of clozapine. Though all these medications individually can cause agranulocytosis, the simultaneous use of all 3 drugs plus a haldol decanoate injection may explain the patient’s absolute neutropenia count of zero. Concurrent valproate use and older age are associated with the development of neutropenia in patients treated with clozapine [10]. 

When severe side effects such as neutropenia or agranulocytosis in TRS prevent the use of clozapine, clinicians must decide whether to re-treat patients with it despite inadequate data. Studies show that rechallenge is helpful, with Dunk et al. reporting that 62% of patients did not develop blood dyscrasia after rechallenge [11,12]. The same study found that 85% of those who experienced blood dyscrasia after a second clozapine trial had severe symptoms and a faster onset. Immediately following the occurrence of neutropenia and agranulocytosis in clozapine-treated patients, the use of G-CSF successfully restores patients' white blood cell count and absolute neutrophil count and shortens the duration of agranulocytosis, according to the research literature [13].

There have been multiple reports on the successful use of granulocyte colony-stimulating factor to treat clozapine-induced neutropenia, but our patient had a delayed response and persisting agranulocytosis with a decreasing ANC until lithium was started as an adjunct to G-CSF and other agranulocytosis-inducing medications were discontinued such as Depakote, making the team more cautious and deciding against clozapine rechallenge [14]. Clozapine was listed as an adverse drug and a drug allergy of our patient due to severe agranulocytosis. Clinicians must weigh the risks and advantages of resuming clozapine, including drug-drug interactions and polypharmacy, even though some cases show rechallenge has worked years after the initial failure [14]. 

Lithium may increase the risk of leukocytosis due to its protective effects on progenitor cells, stimulation of G-CSF production and release, and independent stimulation of progenitor proliferation; thus protective against blood dyscrasias. In light of the leukocytosis effect, it was hypothesized that lithium would be effective in treating clozapine-associated neutropenia [10,15]. While using the promyelocytic side effect of lithium to treat agranulocytosis, other lithium side effects such as tremors, gastrointestinal symptoms, and renal and thyroid effects must be monitored.

## Conclusions

This case shows the synergistic impact of agranulocytosis-inducing drugs, the necessity for clinicians to be cautious when prescribing them concurrently, and the importance of ANC monitoring. We highlighted how lithium can treat G-CSF-resistant or delayed response patients. The prophylactic use of lithium or promyelocytic medications in clozapine use, need further study, especially in individuals with increased susceptibility such as the HLA-DRB1*04:02 allele.

## References

[1] Siskind D, McCartney L, Goldschlager R, Kisely S (2016). Clozapine v. first- and second-generation antipsychotics in treatment-refractory schizophrenia: systematic review and meta-analysis. Br J Psychiatry.

[2] Mijovic A, MacCabe JH (2020). Clozapine-induced agranulocytosis. Ann Hematol.

[3] Li XH, Zhong XM, Lu L (2020). The prevalence of agranulocytosis and related death in clozapine-treated patients: a comprehensive meta-analysis of observational studies. Psychol Med.

[4] Lally J, Malik S, Whiskey E (2017). Clozapine-associated agranulocytosis treatment with granulocyte colony-stimulating factor/granulocyte-macrophage colony-stimulating factor: a systematic review. J Clin Psychopharmacol.

[5] Paton C, Esop R (2005). Managing clozapine-induced neutropenia with lithium. Psychiatri Bull.

[6] Gee S, Vergunst F, Howes O, Taylor D (2014). Practitioner attitudes to clozapine initiation. Acta Psychiatr Scand.

[7] Varghese M T, Jyothi KS, Shaji KS, Rita Venugopal L (2020). Delaying clozapine: how long is too long?. Gen Psychiatr.

[8] Gören JL, Rose AJ, Smith EG, Ney JP (2016). The business case for expanded clozapine utilization. Psychiatr Serv.

[9] Islam F, Hain D, Lewis D, Law R, Brown LC, Tanner JA, Müller DJ (2022). Pharmacogenomics of clozapine-induced agranulocytosis: a systematic review and meta-analysis. Pharmacogenomics J.

[10] Yang CC, Wang XY, Chou PH, Lin CH (2023). Valproate-related neutropenia and lithium-related leukocytosis in patients treated with clozapine: a retrospective cohort study. BMC Psychiatry.

[11] Safferman A, Lieberman J, Alvir JoseMaJ, Howard A (1992). Rechallenge in clozapine-induced agranulocytosis. Lancet.

[12] Dunk LR, Annan LJ, Andrews CD (2006). Rechallenge with clozapine following leucopenia or neutropenia during previous therapy. Br J Psychiatry.

[13] Lam YW (2022). Prolonged agranulocytosis with clozapine despite colony-stimulating factor treatment. Brown Univ Psychopharmacol update.

[14] Özdemir MA, Sofuoğlu S, Tanrikulu G, Aldanmaz F, Eşel E, Dündar S (1994). Lithium-induced hematologic changes in patients with bipolar affective disorder. Biol Psychiatry.

[15] Ghaznavi S, Nakic M, Rao P, Hu J, Brewer JA, Hannestad J, Bhagwagar Z (2008). Rechallenging with clozapine following neutropenia: treatment options for refractory schizophrenia. Am J Psychiatry.

